# Durability of the moderate-to-heavy intensity transition can be predicted using readily available markers of physiological decoupling

**DOI:** 10.1007/s00421-025-05815-0

**Published:** 2025-05-22

**Authors:** Jeffrey A. Rothschild, Gabriele Gallo, Kate Hamilton, Julian D. Stevenson, Harrison Dudley-Rode, Thanchanok Charoensap, Daniel J. Plews, Andrew E. Kilding, Ed Maunder

**Affiliations:** 1 Sports Performance Research Institute New Zealand, Auckland University of Technology, Auckland, New Zealand; 2 High Performance Sport New Zealand, Auckland, New Zealand; 3 Department of Neuroscience, Rehabilitation, Ophthalmology, Genetics, Maternal and Child Health, University of Genoa, Genoa, Italy

**Keywords:** Resilience, Monitoring, Endurance, Cycling

## Abstract

**Purpose:**

To assess relationships between heart rate (HR), ventilation (˙$\dot V$_E_), and respiratory frequency (F_R_) decoupling and durability of the first ventilatory threshold (VT_1_), and the strength of practical models to predict power output at VT_1_ during prolonged exercise.

**Methods:**

Durability of VT_1_ was assessed via measurements of power output at VT_1_ before and after ~ 2.5-h of initially moderate-intensity cycling in 51 trained cyclists, as part of four studies published elsewhere. In 12 of those participants, power output at VT_1_ was assessed every hour until task failure. For every assessment of power output at VT_1_, HR, F_R_, and $\dot V$˙_E_ was measured at fixed power outputs, and thus decoupling of these variables with power output was determined. Bivariate repeated-measures correlations (r_rm_) between decoupling and durability of VT_1_ were assessed. Multivariable models were created to predict power output at VT_1_ during prolonged exercise using generalised estimating equations.

**Results:**

Negative correlations were observed between exercise-induced change in power output at VT_1_ and HR (r_rm_ = −0.76, *P* < 0.001) and F_R_ (r_rm_ = −0.40, *P* = 0.013) decoupling, but not $\dot V$˙_E_ decoupling (r_rm_ = −0.25, *P* = 0.136). The final prediction model, containing baseline VT_1_ and peak oxygen uptake, F_R_ decoupling, and an interaction between HR decoupling and exercise duration, effectively predicted real-time VT_1_ (mean absolute error, ~ 7.2 W; *R*^2^, 0.95).

**Conclusion:**

HR and/or F_R_ decoupling during controlled training sessions may be a practically useful durability assessment. Our prediction models may be an effective means of improving within-session intensity regulation and training load monitoring.

## Introduction

The magnitude of physiological stress responses induced by exercise depends on exercise intensity, and can be categorised into distinct exercise intensity domains. Exercise performed in the moderate-, heavy-, and severe-intensity domains is characterised by attainment of a rapid steady state, delayed steady state, or absence of a steady state, respectively, in physiological variables such as oxygen consumption ($\dot V$O_2_), blood lactate concentration, and blood and muscle pH (Black et al. 2017; Jones et al. 2008). Accordingly, training is often programmed and monitored in accordance with estimates of work outputs at the intensity domain transitions, derived from laboratory or field-based physiological profiling assessments (Jamnick et al. 2020; Maunder et al. 2021; Seiler 2010). The moderate-to-heavy intensity transition is typically estimated using the first ventilatory threshold (VT_1_) or lactate threshold method, in which the intensity at which the ventilatory equivalent for oxygen or blood lactate concentration starts to rise above baseline levels (Jamnick et al. 2020), whilst the heavy-to-severe intensity transition is typically estimated using the power/speed-duration relationship of maximum lactate steady state methods (Jones et al. 2019). Power output at the moderate-to-heavy (Stevenson et al. 2022; Gallo et al. 2024; Hamilton et al. 2024; Dudley-Rode et al. 2024) and heavy-to-severe (Clark et al. 2018, 2019a, 2019b; Spragg et al. 2023) intensity transitions decreases in a non-linear fashion during prolonged exercise. ‘Durability’ refers to an athlete’s resilience to the negative effects of prolonged exercise on the intensity domain transitions. (Maunder et al. 2021).

Inter-individual differences in durability complicate the translation of physiological profiling data to real-world training programming and load monitoring. For example, following 2.5 h of moderate-intensity exercise, we have observed decreases in power output at the moderate-to-heavy intensity transition ranging from 1 to 45 W (Stevenson et al. 2022; Hamilton et al. 2024; Dudley-Rode et al. 2024). This is problematic as coaches may programme training sessions in relation to baseline values of power output at an intensity domain transition, and large reductions in power output at the intensity domain transition during exercise could lead to a dissociation between the planned and achieved physiological demands on the athlete. Furthermore, given the emerging evidence that durability is an influential performance determinant (Gallo et al. 2024; Hamilton et al. 2024), development of field-based assessments of durability are warranted (Maunder et al. 2021). Identifying a metric that acknowledges durability and can be used for real-time assessment of proximity to intensity domain transitions during prolonged exercise without invasive physiological measures or laboratory assessments, as well as for accurate training load monitoring, is also desirable.

Decoupling of physiological variables, such as heart rate, from external work outputs during prolonged exercise has been used to make inferences about durability (Maunder et al. 2021; Smyth et al. 2022) and measures of within-session decoupling are calculated on some endurance training software platforms (e.g. TrainingPeaks, Boulder, CO, USA). However, relationships between common decoupling measures and durability have not previously been assessed. It is, therefore, unclear if decoupling reflects loss of power output at the intensity domain transitions, and/or if decreased decoupling in response to the same training session reflects improved durability (Maunder et al. 2021). Accordingly, assessment of the ability of various decoupling measures to predict durability is warranted.

Therefore, the aim of the present investigation was to (i) assess bivariate relationships between decoupling of heart rate, ventilation, and breathing frequency and durability of power output at the moderate-to-heavy intensity transition and (ii) assess the strength of practical models including decoupling measures to predict power output at the moderate-to-heavy intensity transition at multiple time points during prolonged exercise. We hypothesised that the magnitude of decoupling between heart rate, ventilation, and breathing frequency and power output would be associated with the durability of the moderate-to-heavy intensity transition, and that practical models including these decoupling measures would accurately predict power output at the moderate-to-heavy intensity transition in real-time. We combined data from multiple recent studies from our laboratory assessing the durability of the moderate-to-heavy intensity transition to perform these analyses. (Stevenson et al. 2022; Gallo et al. 2024; Hamilton et al. 2024; Dudley-Rode et al. 2024).

## Methods

### Ethical approval

The study was performed in accordance with the Declaration of Helsinki, 2013. The Auckland University of Technology Ethics Committee approved all procedures (21/253, 22/163, 23/37, 23/197), and all participants provided written informed consent prior to participation. This study was not registered in a database. Raw data is available upon request.

### Study design

**Fig. 1:**
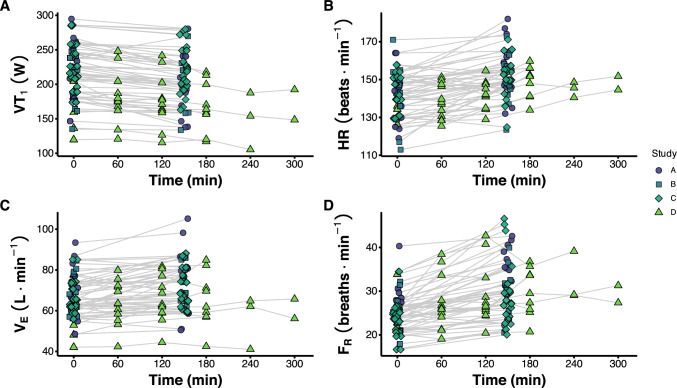
Overview of power at the first ventilatory threshold (VT_1_) across all time points from each study included in the analysis

This study features data from 51 unique trained cyclists, collected as part of four studies published elsewhere (Stevenson et al. 2022; Gallo et al. 2024; Hamilton et al. 2024; Dudley-Rode et al. 2024). Accordingly, no specific sample size estimate was made for this exploratory analysis. Power output at the moderate-to-heavy intensity transition was estimated before and after ~ 2.5-h of initially moderate-intensity cycling using an incremental exercise assessment with indirect calorimetry in each participant. In 12 participants, power output at the moderate-to-heavy intensity transition was estimated every hour until task failure. For every estimate of power output at the moderate-to-heavy intensity transition, heart rate (HR), respiration frequency (F_R_), and minute ventilation ($\dot V$˙_E_) was measured at fixed power outputs, and thus decoupling of these variables with power output was determined concomitant with changes in power output at the moderate-to-heavy intensity transition (Fig. 1).

### Experimental protocols

All participants initially reported to the laboratory following an overnight fast. After providing informed consent and completing a health screening questionnaire, height and body mass was measured. Subsequently, participants undertook a maximal, incremental cycling test on a laboratory ergometer (Stevenson et al. 2022) or personal road bicycle mounted to a direct-drive indoor cycling smart trainer (Kickr, Wahoo Fitness, GA, USA) (Gallo et al. 2024; Hamilton et al. 2024; Dudley-Rode et al. 2024). The purpose of this initial visit was to estimate peak oxygen uptake ($\dot V$O_2_peak) and power output at the first ventilatory threshold (VT_1_) for the design of the subsequent exercise protocols. The maximal, incremental test began at 95 W, and power output increased by 35 W every 3 min until attainment of the second ventilatory threshold (VT_2_) was confirmed by a clear rise in the ventilatory equivalent for CO_2_ and reduction in the partial pressure of end-tidal CO_2_ (Jamnick et al. 2020). Subsequently, power output was increased by 35 W every minute until task failure. Expired gases and heart rate were collected continuously using indirect calorimetry (TrueOne 2400, ParvoMedics, UT, USA) and a chest-strap heart rate monitor (Polar Electro Oy, Kempele, Finland). The $\dot V$O_2_peak was quantified as the highest 15-s average $\dot V$O_2_. The VT_1_ was identified as the first $\dot V$O_2_ vs. $\dot V$_E_^.^$\dot V$O_2_^−1^ relationship (Jamnick et al. 2020). This $\dot V$O_2_ was converted to power output by linear regression of the $\dot V$O_2_ vs. power output relationship, using the last minute of $\dot V$O_2_ data from each 3-min stage. The VT_2_ was quantified using the same method, where the breakpoint in the $\dot V$O_2_ vs. $\dot V$_E_^.^V̇CO_2_^−1^ relationship was identified as VT_2_.

One cohort of 14 participants reported to the laboratory for one further laboratory visit following the maximal, incremental test assessment, again after an overnight fast and having consumed ~ 800 mL of water one hour beforehand (Study A) (Stevenson et al. 2022). This second visit involved assessment of power output at the moderate-to-heavy intensity transition using an individualised five-step incremental test performed before and after 2-h of cycling at 90% of the power output at VT_1_ estimated in the first visit. Participants consumed plain water ad libitum during the 2-h prolonged cycling phase. The five-step test took 30 min, and hence the second five-step test took place after 2.5 h of exercise had already been completed. Following a 5-min warm-up at 100 W, the five-step incremental test began 50 W below power output at VT_1_ estimated in the first visit, and increased by 25 W every 4 min, such that the fifth and final step was 50 W above the power output at VT_1_ estimated in the first visit. The five-step test was followed by 5 min at 100 W. Expired gases and heart rate were collected continuously using indirect calorimetry (TrueOne 2400, ParvoMedics, UT, USA) and a chest-strap heart rate monitor (Polar Electro Oy, Kempele, Finland). Power output at the moderate-to-heavy intensity transition was estimated using the VT_1_ method according to the methods of the first laboratory visit, where $\dot V$O_2_ data from the last minute of each 4-min step was used to create the $\dot V$O_2_ vs. power output relationship, and compared between the five-step incremental tests performed before and after the 2 h of moderate-intensity cycling. The average HR, $\dot V$_*E*_, and F_R_ for the last 1-min of each 4-min step was also quantified. Decoupling was quantified as the difference between the HR, $\dot V$˙_E_, and F_R_ in the third step of the five-step incremental tests performed before and after 2-h of moderate-intensity cycling. Change in power output at the moderate-to-heavy intensity transition and decoupling was therefore quantified once for all participants with one missing data point (due to technical errors during the incremental test), and therefore these data contributed 13 datapoints to the present analysis.

One cohort of 13 participants reported to the laboratory for two further laboratory visits following the maximal, incremental test assessment, having consumed a breakfast containing ~ 2 g^.^kg^−1^ of carbohydrate and ~ 800 mL of water one hour beforehand (Study B) (Hamilton et al. 2024). These two visits occurred in random order. These visits involved assessment of power output at the moderate-to-heavy intensity transition and HR, F_R_, $\dot V$_E_ decoupling using the five-step incremental test described above. The five-step incremental test was performed with and without 2.5 h of prior cycling at 90% of the power output at VT_1_ estimated in the first visit, during which participants consumed 150 mL of water every 15 min in a solution made with electrolyte mix (LMNT, 125 mg Na^+^, 25 mg K^+^, and 7.5 mg Mg^2+^) during the first 120 min. Power output at the moderate-to-heavy intensity transition was compared between the five-step incremental tests performed with and without 2.5 h of prior moderate-intensity cycling. Decoupling was quantified as the difference between the average HR, $\dot V$˙_E_, and F_R_ in the last 1-min of the third step of the five-step incremental tests performed with and without 2.5 h of moderate-intensity cycling. Change in power output at the moderate-to-heavy intensity transition and decoupling was therefore quantified once for all participants. As two participants had missing ventilatory data, these data contributed 11 datapoints to the present analysis.

One cohort of 12 participants reported to the laboratory for three further laboratory visits following the maximal, incremental test assessment, having consumed a breakfast containing ~ 1 g^.^kg^−1^ of carbohydrate and ~ 800 mL of water one hour beforehand (Study C) (Dudley-Rode et al. 2024). These three visits occurred in random order. These visits involved assessment of power output at the moderate-to-heavy intensity transition and HR, F_R_, $\dot V$˙_E_ decoupling using the five-step incremental test procedures described above. The five-step incremental test was performed twice with and once without 2.5 h of prior cycling at 90% of the power output at VT_1_ estimated in the first visit. Participants consumed either 120 g of glucose or a sweetened placebo during the first 120 min of the 2.5 h of moderate-intensity cycling (60 g^.^h^−1^). Power output at the moderate-to-heavy intensity transition was compared between the five-step incremental test performed without 2.5 h of prior moderate-intensity cycling and the two five-step incremental tests performed following 2.5 h of prior moderate-intensity cycling. Decoupling was quantified as the difference between the HR, $\dot V$_E_, and F_R_ in the last 1-min of the third step of the five-step incremental test performed without 2.5 h of moderate-intensity cycling and the two five-step incremental tests performed following 2.5 h of prior moderate-intensity cycling. Change in power output at the moderate-to-heavy intensity transition and decoupling was therefore quantified twice for all participants with one missing datapoint (one participant dropped out of the study before completing the prolonged trial with carbohydrate ingestion), and therefore these data contributed 23 datapoints to the present analysis.

One cohort of 12 participants reported to the laboratory for one further laboratory visit following the maximal, incremental test assessment, having consumed a breakfast containing ~ 2 g^.^kg^−1^ of carbohydrate and ~ 800 mL of water one hour beforehand (Study D) (Gallo et al. 2024). This second visit involved assessment of power output at the moderate-to-heavy intensity transition using an individualised six-step incremental test every hour. Following a 3-min warm-up at 100 W, the six-step incremental test began at a power output 30% lower than VT_1_ estimated in the first visit, and increased by 10% of the power output at VT_1_ estimated in the first visit every 4 min, such that the sixth and final step was 20% above the power output at VT_1_ estimated in the first visit. The six-step step was followed by 3 min at 100 W, and then 30 min at 90% of the power output at VT_1_ estimated in the first visit, such that power output at the moderate-to-heavy intensity transition and HR, $\dot V$˙_E_, and F_R_ decoupling was measured every hour. These protocols were repeated until task failure, to a maximum of 6 h (234 ± 66 min). Power output at the moderate-to-heavy intensity transition was estimated using the VT_1_ method according to the methods described above, and compared between the first and all subsequent six-step incremental tests. The HR, $\dot V$_E_, and F_R_ during each 4-min step were also quantified. Decoupling was quantified as the difference between the average HR, $\dot V$E, and F_R_ in the last 1-min of the fourth step during the first and all subsequent six-step incremental tests. Change in power output at the moderate-to-heavy intensity transition and decoupling was therefore quantified at multiple timepoints for all participants, and therefore these data contributed 38 datapoints to the present analysis.

### Data analysis

Exercise-induced changes in power output at the moderate-to-heavy intensity transition, and HR, $\dot V$_E_, and F_R_ at a fixed power output, was therefore quantified for 48 participants at 85 timepoints, by subtraction of the value derived from the five- or six-step incremental test performed without prior prolonged exercise from each five- or six-step incremental test performed with prior prolonged exercise.

### Statistical analysis

Bivariate relationships between durability of the moderate-to-heavy intensity transition (VT_1_) and HR, $\dot V$_E_, and F_R_ decoupling were assessed using repeated-measures correlation, which allows analysis of repeated measures data without violating independence assumptions (Bakdash and Marusich 2017). One data point was deemed an outlier to and removed from the modelling analysis based on median absolute deviation calculations (Leys et al. 2019), combined with the physiological implausibility of increasing power output at the moderate-to-heavy intensity transition by 29 W following 150 min of cycling.

Multivariable models were created to predict power output at the moderate-to-heavy intensity transition following exercise of varying durations using generalised estimating equations (GEE). GEE models provide population-averaged (e.g., marginal), rather than subject-specific models while accounting for repeated measurements within participants (McNeish 2023). Quasi Information Criterion (QIC) was used for selecting an independence correlation structure as the working correlation matrix (Pan 2001). The following variables were considered for the full models: power output at VT_1_ assessed in the initial visit, HR decoupling, HR decoupling squared, F_R_ decoupling, F_R_ decoupling squared, V̇_E_ decoupling, exercise time (min), $\dot V$O_2_peak assessed in the initial visit (mL^.^kg^−1.^min^−1^), absolute$\dot V$ O_2_ at VT_2_ assessed in the initial visit (L^.^min^−1^), relative $\dot V$O_2_ at VT_2_ assessed in the initial visit (% of $\dot V$O_2_peak), power output at VT_2_ assessed in the initial visit (W), relative power output at VT_2_ assessed in the initial visit (W^.^kg^−1^), and self-reported typical weekly training volume (h). In addition, interactions between F_R_ decoupling and time, HR decoupling and time, relative $\dot V$O_2_peak and time, and relative $\dot V$O_2_peak and baseline VT_1_ power were considered in the full model. The top candidate models were identified using the glmulti R package (Calcagno and Mazancourt 2010) which performs a genetic search across possible models specified by a given set of predictors and selects the top models according to corrected Akaike Information Criterion (AICc), set to a maximum model size of five predictors. From the reduced pool of models, we performed 2000 bootstrap resamples (Collins et al. 2024) using the Tidymodels ecosystem in R, selecting the model with the lowest mean absolute error (MAE) as the final model. If two models had similar performance, the one with fewer predictor variables was chosen. Due to the limited access to F_R_ data in practical settings, we also included models with easily obtainable measures ($\dot V$O_2_peak and HR). The fit of each model was checked by visualizing the Q–Q and other residual plots to ensure approximate residual normality and homoscedasticity using the performance R package. Model performance is reported as the coefficient of determination (*R*^2^), which represents the proportion of variance explained by the model, and the MAE, which quantifies the average absolute discrepancy between the observed and predicted values. These metrics were calculated using both in-sample data (i.e., the same data used to train the model and evaluate performance) and using bootstrap resampling, which offers a more realistic and unbiased (or least biased) estimate of model performance in the population in which the model is intended (Collins et al. 2024). All analyses were carried out with R version 4.3.1 (The R foundation for Statistical Computing, Vienna, Austria).

Future studies could validate these models in other groups of cyclists, following different protocols. Based on the approach of Archer et al. (Archer et al. 2021), it can be suggested that a minimum sample size of 22 subjects would be needed in a validation dataset to obtain precise estimates of *R*^2^ (proportion of variance in explained in the external validation dataset), calibration in the large (the difference between mean observed and mean predicted outcome values), and calibration slope (the agreement between predicted and observed values across the range of predicted values), assuming 95% confidence intervals with target widths of 0.1 for *R*^2^ and 0.2 for calibration slope (see supplemental R code).

## Results

Participant characteristics for each group are shown in Table 1. An overview of the power output at VT_1_ across all time points is shown in Fig. 1. There are 48, 12, 12, 47, 9, 3, and 2 data points at minutes 0, 60, 120, 150, 180, 240, and 300, respectively.

**Table 1:** Participant characteristics

Study	N (m/f)	Age (y)	$\dot V$˙O_2_peak (mL^.^kg^−1.^min^−1^)	VT_1_ (W)	VT_2_ (W)	Training volume (h^.^week^−1^)
A^7^	13/1	34 ± 10	59.9 ± 6.8	217 ± 42	275 ± 51	9 ± 3
B^9^	11/2	29 ± 8	57.4 ± 5.2	211 ± 40	274 ± 53	12 ± 2
C^10^	10/2	31 ± 6	59.3 ± 5.3	229 ± 37	290 ± 38	14 ± 5
D^8^	10/2	41 ± 8	52.3 ± 5.2	184 ± 36	256 ± 48	10 ± 3

Values shown as mean ± SD

**Fig. 2:**
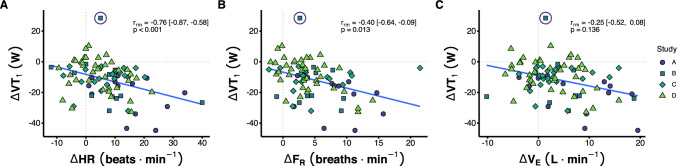
Bivariate linear repeated measures correlations between the exercise-induced change in power output at VT_1_ (W) and (**a**) the exercise-induced change in heart rate (HR, beats^.^min ^−1^), (**b**) the xercise-induced change in respiratory frequency (F_R_, breaths^.^min^−1^), and (**c**) the exercise-induced change in ventilation rate ($\dot V$_E_, L^.^min^−1^), coloured by study. Outliers not included in the statistical analysis are circled

Negative correlations were observed between the exercise-induced change in power output at VT_1_ (W) and the change in HR (beats^.^min^−1^, r_rm_ = −0.76 [95% CI −0.87, -0.58], *P* < 0.001) and the change in F_R_ (breaths^.^min^−1^, r_rm_ = −0.40 [−0.64, −0.09], *P* = 0.013). However, the relationship between the exercise-induced change in power output at VT_1_ (W) and the change in $\dot V$_E_ (L^.^min^−1^) was not significant (r_rm_ = −0.25 [−0.52, 0.08], *P* = 0.136, Fig. 2).

**Fig. 3:**
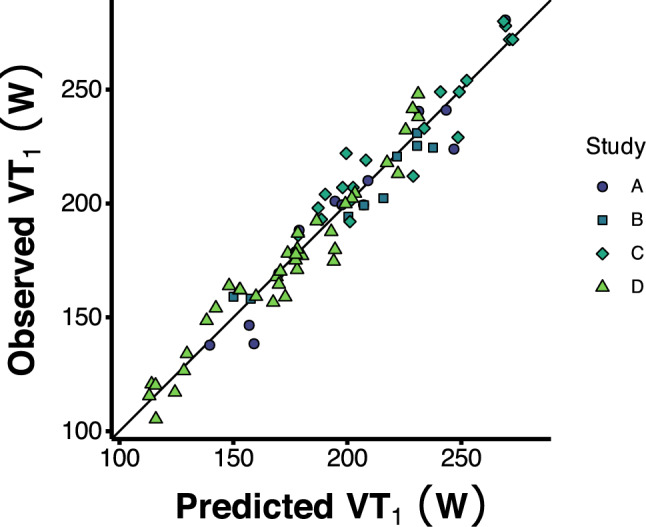
Predicted vs. observed values for power at the moderate-to-heavy intensity transition (VT_1_), coloured by study

**Fig. 4:**
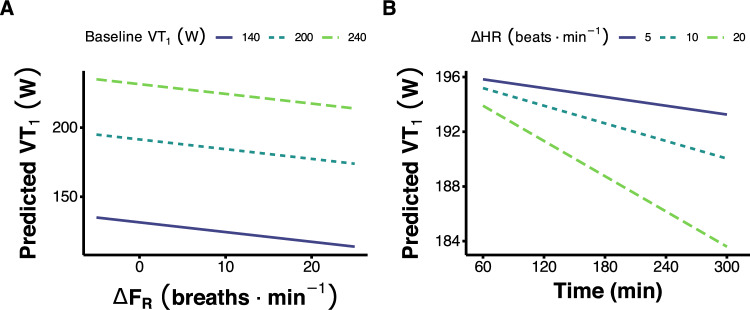
Model predictions showing the expected values for VT_1_ across a range of values for change in respiratory frequency (F_R_, panel A), or time (panel B), with the different lines depicting a different baseline value for VT_1_ (panel A), or change in heart rate (HR, panel B). The differing slopes of the lines in (B) illustrate the interaction term between the change in HR and time included in the model

The final prediction models are shown in Table 2. Model coefficients are provided, along with model performance metrics *R*^2^ and MAE using both in-sample data and bootstrap cross-validation. These models explained a large amount of variance (*R*^2^ values of 0.93–0.95) and displayed good accuracy (MAE of 7.2–8.7 W). For the best-performing model, visual depiction of the predicted vs. observed values is shown in Fig. 3. Model predictions showing the expected values for VT_1_ across a range of values for change in F_R_ or time are shown in Fig. 4. The interaction between change in HR and time is illustrated in Fig. 4B, as changes in HR are associated with greater reductions in VT_1_ power as exercise duration extends.

**Table 2:** Model performance and coefficients

Model	MAE	MAE-cv	*R*^2^	*R*^2^-cv	Intercept	Baseline VT_1_	ΔF_R_	ΔHR:Time	$\dot V$˙O_2_peak	ΔHR
Baseline VT_1_ + ΔF_R_ + ΔHR:Time + $\dot V$O_2_peak	7.2	8.3 8.3, 8.4]	0.95	0.93 [0.93, 0.93]	21.7 [0.31, 46.3]	1 [0.91, 1.1]	−0.58 [−1.4, 0.11]	−0.003 [−0.006, −0.002]	−0.53 [−1.3, 0.11]	–
Baseline VT_1_ + ΔHR + $\dot V$_2_peak	7.6	8.4 [8.3, 8.5]	0.94	0.93 [0.93, 0.93]	22.5 [−3.6, 52.0]	1 [0.93, 1.10]	–	–	−0.56 [−1.40, 0.04]	−0.91 [−1.60, −0.37]
Baseline VT_1_ + ΔHR	8.3	8.7 [8.6, 8.7]	0.94	0.93 [0.93, 0.93]	1.6 [−9.0, 15.4]	0.96 [0.89, 1.00]	–	–	–	−0.75 [−1.60, −0.38]

^1^Values shown as mean [95% confidence intervals] from 2000 bootstrap resamples. F_R_ = respiratory frequency, MAE = Mean Absolute Error, MAE-cv = MAE from bootstrap resampling, *R*^2^-cv = R^2^ from bootstrap resampling

## Discussion

The key findings from this study are: (i) significant negative relationships were observed between exercise-induced changes in power output at the moderate-to-heavy intensity transition and HR and F_R_ decoupling, but not $\dot V$˙_E_ decoupling, and (ii) prediction models that include HR and F_R_ decoupling along with measures of baseline fitness can provide practically meaningful insight into dynamic changes in power output at the moderate-to-heavy intensity transition during prolonged cycling. These findings have implications for durability assessment, within-session intensity regulation, and training programming and load monitoring in endurance sport.

Our observations that HR and F_R_ decoupling are significantly correlated with durability of the moderate-to-heavy intensity transition has practical implications (Fig. 2). Our data suggest that the magnitude of HR or F_R_ decoupling during controlled training sessions may be a practical method of durability assessment, and serial measures over time may be a useful means of monitoring durability-related adaptations. The greater strength of the relationship between HR decoupling and the moderate-to-heavy intensity transition vs. F_R_ decoupling suggests HR decoupling may be more useful for this purpose. This supports research in which HR decoupling has been used to group athletes into more and less durable subgroups (Maunder et al. 2021; Smyth et al. 2022). Our previous research suggested that programming and regulating training in accordance with the HR observed at the baseline moderate-to-heavy intensity transition fails to appreciate the dynamic nature of the intensity domain transitions during prolonged exercise (Stevenson et al. 2022). Indeed, across our whole cohort, HR at VT_1_ after 150/180 min of cycling was significantly elevated compared to the baseline value, despite the reduction in power output at VT_1_ (N = 58, 142 ± 11 vs. 147 ± 10 b^.^min^−1^, *P* = 0.0002). Therefore, whilst a stable baseline HR threshold value cannot be used to assess proximity to the moderate-to-heavy intensity transition in real-time during prolonged exercise, these data suggest more durable athletes exhibit less HR decoupling, and therefore HR decoupling during controlled, prolonged exercise may be a useful durability assessment.

The absence of a significant relationship between the magnitude of $\dot V$_E_ decoupling and durability of the moderate-to-heavy intensity transition is surprising. The $\dot V$_E_ is sensitive to a range of metabolic inputs including stimulation of central and peripheral chemoreceptors, skeletal muscle metaboreceptors, and exercise-induced changes in CO_2_, pH, and skeletal muscle metabolites (Nicolò et al. 2020; Tipton et al. 2017). Also, we and others have observed that $\dot V$_E_ at VT_1_ remains relatively stable during prolonged exercise (Stevenson et al. 2024; Nuuttila et al. 2024), and therefore suggested that real-time monitoring of $\dot V$˙_E_ may be a useful means of assessing proximity to the moderate-to-heavy intensity transition during prolonged exercise. We therefore hypothesised that those exhibiting the largest reductions in power output at the moderate-to-heavy intensity would also see the largest magnitude of $\dot V$_E_ decoupling, given the differences in muscle and systemic metabolite accumulation during exercise in the moderate- and heavy-intensity domains (Black et al. 2017). The physiological regulation of $\dot V$_E_ is complicated, with emerging evidence for differential regulation of F_R_ and tidal volume provided in studies of ventilatory responses to rapid changes in exercise work rate (Nicolò et al. 2017, 2018), visualisation of exercise at rest (Wuyam et al. 1995; Decety et al. 1993), or under hypnosis (Thornton et al. 2001), and exercise in hypercapnia (Clark et al. 1980). Our results suggest that while the $\dot V$_E_ associated with the moderate-to-heavy intensity transition may remain stable during prolonged exercise, the magnitude of $\dot V$_E_ drift does not seem to provide insight into the magnitude of the loss of power output at the moderate-to-heavy intensity transition, at least in this dataset. The reason for this discrepancy is not immediately clear, and may be related to noise in the datasets, as the slope of the line-of-best-fit is similar to those between loss of power output at the moderate-to-heavy intensity transition and HR and F_R_ drift (Fig. 2). Similarly, it is possible that a relationship between $\dot V$˙_E_ decoupling and durability might have emerged in more-demanding exercise protocols, as the noise associated with the measurement of $\dot V$˙_E_ drift may have been proportionally less of the observed effects. More investigation of the utility of $\dot V$_E_ drift in real-time assessment of power output at the intensity domain transitions during prolonged exercise is warranted.

The second aim of the study was to model and predict power output at the moderate-to-heavy intensity transition at multiple time points during prolonged cycling. Addressing this aim has practical implications, as many coaches programme training based on power output observed at the intensity domain transitions. Therefore, failing to account for prolonged exercise-induced reductions in power output at the intensity domain transitions (Stevenson et al. 2022; Gallo et al. 2024; Hamilton et al. 2024; Clark et al. 2018, 2019a, 2019b) could lead to a dissociation between the planned and achieved physiological demands on the athlete (Maunder et al. 2021). Accordingly, identification of tools that can be used to assess dynamic changes in power output at the intensity domain transitions would allow more physiologically informed within-session intensity regulation, and more accurate quantification of training intensity distribution and training load.

The best model was selected from a pool of models containing the three decoupling measures, along with commonly used and easily obtainable profiling measures such as $\dot V$O_2_peak and baseline ventilatory thresholds. The final model included F_R_ decoupling, an interaction between HR decoupling and time, $\dot V$O_2_peak (mL^.^kg^−1.^min^−1^), and baseline power output at VT_1_. We had hypothesised that these factors would contribute to model performance, as they reflect the influence of both baseline fitness and the physiological effects of prolonged exercise. Using internal validation (i.e., testing the model on the same data that was used to train the model), *R*^2^ was 0.95 and MAE was 7.2 W. This is referred to as ‘apparent performance’, which typically provides overly optimistic values compared with when the model is evaluated in new data (Steyerberg and Harrell 2016). Therefore, it is recommended that prediction models should have additional internal–external, and/or external validation (Steyerberg and Harrell 2016). Internal–external validation refers to using a portion of the data for training a model and a separate portion of data for testing it. We used the bootstrap resampling approach, which means repeatedly drawing samples with replacement from the original dataset to create multiple training and testing sets (Efron and Tibshirani 1986). This allows for the estimation of the sampling distribution of the model’s performance metrics and provides more robust measures of accuracy, including confidence intervals for the model’s predictions (Steyerberg and Harrell 2016). Using this resampling approach, *R*^2^ values were very stable at 0.93 [95% CI 0.93–0.93], and MAE was also stable (8.3 [8.3–8.4] W). This represents a very small yet expected decline in performance from the apparent performance (Table 2). This average prediction error in our models is also within the previously reported 3.5% coefficient of variation for power at VT_1_ (Pallarés et al. 2016). This also highlights the utility of our models, as meaningful changes (i.e., beyond day-to-day measurement error) should be detected. In addition, our simpler models, which may be more practical in some applied settings, also displayed good performance (Table 2).

The ability of our model to accurately predict dynamic changes in power output at the moderate-to-heavy intensity transition in real-time during prolonged exercise is useful as a proof-of-concept. Being able to accurately predict power output at the intensity domain transitions in real-time during prolonged exercise may allow an athlete to more accurately regulate intensity within sessions, and therefore more accurately adhere to an intensity domain-specific training prescription (e.g., 3 h in the moderate-intensity domain, with specific intervals in the heavy-intensity domain). This may allow for more effective management of training stress. Similarly, being able to estimate changes in power output at the intensity domain transitions that occurred within individual sessions may allow for more physiologically informed quantification of training load and training intensity distribution. Future studies using similar methods could develop models to accurately predict dynamic changes in power output at the heavy-to-severe intensity transition during prolonged exercise, to further support applied practice.

There are, however, several limitations that should be noted. All studies used in our analysis involved prolonged exercise (primarily) at 90% of the initial power output at the moderate-to-heavy intensity transition, and so we cannot determine if our models would be as effective when applied to prolonged exercise at different intensities or intermittent exercise. In addition, pre- and peri-exercise feeding was controlled in each of the studies included in the analysis, and for a model to be usable in the real-world, it needs to be robust to the diverse range of pre- and peri-exercise nutrition strategies used by athletes. These limitations notwithstanding, our data provide a useful proof-of-concept that models including physiological decoupling and baseline descriptive characteristics can be developed to predict dynamic changes in power output at the moderate-to-heavy intensity transition during prolonged exercise.

In summary, in this secondary analysis of data from four of our previous studies, we observed significant negative relationships between exercise-induced changes in power output at the moderate-to-heavy intensity transition and HR and F_R_, but not ˙$\dot V$_E_, decoupling, and that prediction models including HR and F_R_ decoupling and measures of baseline fitness can accurately predict dynamic changes in power output at the moderate-to-heavy intensity transition during prolonged cycling. These observations suggest that quantification of HR decoupling during controlled training sessions may be a practically useful durability assessment, and that prediction models may be an effective means of improving within-session intensity regulation and training load monitoring.

## Abbreviations

F_R_: Respiratory frequency
HR: Heart rate
V_*T*_: Tidal volume
$$\dot V$$˙_E_: Minute ventilation
$\dot V$O_2_: Rate of oxygen consumption
$\dot V$O_2_peak: Peak rate of oxygen consumption
VT_1_: First ventilatory threshold
VT_2_: Second ventilatory threshold

## Declarations

### Data availability

Data is available from the corresponding author upon reasonable request.

### Code availability

Code is available upon request.
